# Quantitative profiling of *N*^6^-methyladenosine at single-base resolution in stem-differentiating xylem of *Populus trichocarpa* using Nanopore direct RNA sequencing

**DOI:** 10.1186/s13059-020-02241-7

**Published:** 2021-01-07

**Authors:** Yubang Gao, Xuqing Liu, Bizhi Wu, Huihui Wang, Feihu Xi, Markus V. Kohnen, Anireddy S. N. Reddy, Lianfeng Gu

**Affiliations:** 1 Basic Forestry and Proteomics Research Center, College of Life Science, Fujian Provincial Key Laboratory of Haixia Applied Plant Systems Biology, Fujian Agriculture and Forestry University, Fuzhou, 350002 China; 2 Basic Forestry and Proteomics Research Center, College of Forestry, Fujian Agriculture and Forestry University, Fuzhou, 350002 China; 3 Department of Biology and Program in Cell and Molecular Biology, Colorado State University, Fort Collins, CO USA

**Keywords:** *N*^6^-Methyladenosine, Nanopore direct RNA sequencing, Alternative polyadenylation, Stem-differentiating xylem, *Populus trichocarpa*

## Abstract

There are no comprehensive methods to identify *N*^6^-methyladenosine (m^6^A) at single-base resolution for every single transcript, which is necessary for the estimation of m^6^A abundance. We develop a new pipeline called Nanom6A for the identification and quantification of m^6^A modification at single-base resolution using Nanopore direct RNA sequencing based on an XGBoost model. We validate our method using methylated RNA immunoprecipitation sequencing (MeRIP-Seq) and m^6^A-sensitive RNA-endoribonuclease–facilitated sequencing (m6A-REF-seq), confirming high accuracy. Using this method, we provide a transcriptome-wide quantification of m^6^A modification in stem-differentiating xylem and reveal that different alternative polyadenylation (APA) usage shows a different ratio of m^6^A.

**Supplementary Information:**

The online version contains supplementary material available at 10.1186/s13059-020-02241-7.

## Introduction

*N*^6^-Methyladenosine (m^6^A) in plants is the most prevalent RNA modification [1], which is implicated in regulating many aspects of gene regulation and development [2, 3]. Methylated RNA immunoprecipitation sequencing (MeRIP-Seq/m^6^A-seq) has been useful in unveiling m^6^A-enriched transcripts in *Arabidopsis thaliana* [4]. Variations in m^6^A-seq such as photo-crosslinking-assisted m^6^A-sequencing (PA-m^6^A-seq) in humans [5], m^6^A-crosslinking immunoprecipitation (m^6^A-CLIP) associated with crosslinking-induced mutation sites (CIMS) in mouse [6], and m^6^A individual-nucleotide-resolution crosslinking and immunoprecipitation (miCLIP) in humans [7] have been used to detect m^6^A sites at single-base resolution. However, these methods are challenging in plants due to the low efficiency of UV crosslinking and the time required to construct highly complex libraries. Recently, antibody-independent quantitative profiling of m^6^A sites has been reported [8, 9]. The RNA digestion via m^6^A-sensitive enzyme method called m^6^A-REF-seq [9] or MAZTER-seq [8] can identify and quantify RNA modification in the ACA motif at single-nucleotide resolution. However, other known DRACH (D = G/A/U, R = G/A, H = A/U/C) motif would be missed since the enzyme digestion-based method recognizes only ACA sites.

The direct RNA sequencing (DRS) technique developed recently by Oxford Nanopore Technologies (ONT) has the potential to detect base modification signals in RNA [1]. Both *EpiNano* [10] and MINES (m^6^A Identification using Nanopore Sequencing) [11] provide a valuable qualitative profile of m^6^A sites based on DRS. *EpiNano* can detect modified bases through increased mismatches around the modified RNA base due to decreased basecalling qualities using support vector machines (SVM) [10]. However, the algorithm of *EpiNano* could not distinguish m^6^A from other types of RNA modification, such as m^1^A [10]. MINES can detect m^6^A sites in only four types of sequence context (AGACT, GGACA, GGACC, and GGACT) through fraction modification values with random forest (RFM) method using methylation sites from miCLIP data [11]. Moreover, a quantitative comparison of m^6^A in different samples is especially important to gain functional insights about this modification.

It is intuitive to use ionic current signals from the sequencer to identify the base modification. Recently, several pioneering studies adopted this principle. For example, the Gaussian mixture model has been reported to determine unsupervised modification number [12]. Another study on 16S ribosomal RNA reported the correlation between basecall error and ionic current deviations from direct nanopore sequencing [13]. In vitro transcribed RNAs in the GGACU motif also further validated the correlation between m^6^A modification and ionic current signals [14]. MINES also built motif-specific models based on pore current value [11]. These new methods proved that de novo identification of m^6^A is feasible. In this study, we used ionic current signals directly and developed an XGBoost model for the identification of m^6^A modification at a single-base resolution at an individual transcript level, which is necessary for estimation of m^6^A abundance and quantitative comparison of this modification across different samples.

## Results and discussion

### Development of an m^6^A prediction model using XGBoost algorithm based on signals in Nanopore DRS reads

**Fig. 1:**
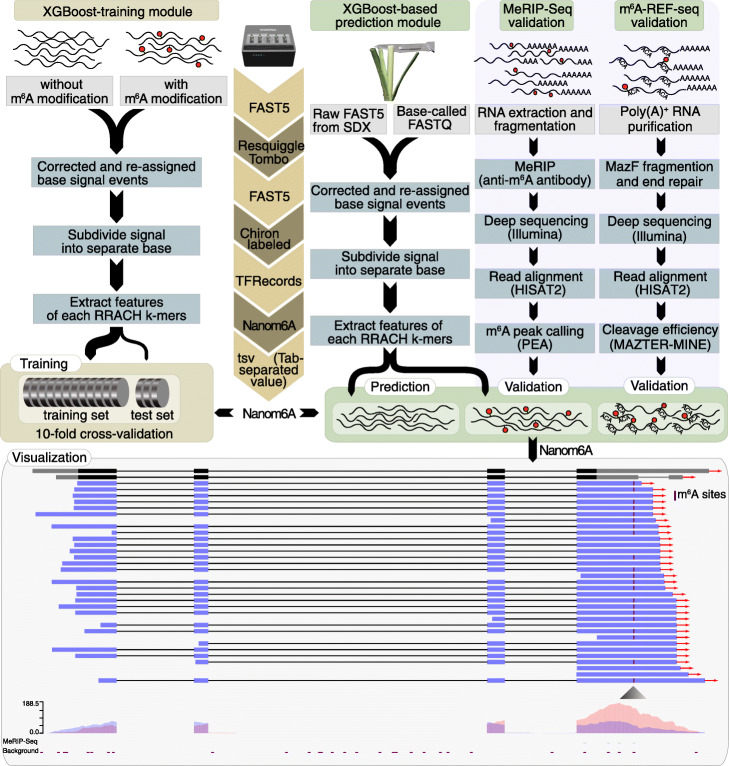
Nanom6A pipeline for identification of m^6^A at single-base resolution. For the XGBoost-training module, the raw sequence with or without m^6^A modification was resquiggled. After the corrected sequence was assigned to the raw signal using Tombo, we extracted the signals of separate bases according to RRACH features including signal length, mean, std., and width. The corresponding 12 k-mers were trained to build the XGBoost models separately. For the prediction module, the basecalled files were aligned to the genome sequence. Then, the sequence was corrected with the genome sequence. After the corrected sequence was assigned to the raw signal, signals of individual bases, corresponding features of RRACH k-mer were extracted to predict the modified bases with the trained models

Here we developed a new pipeline called Nanom6A to identify and visualize m^6^A sites at a single-nucleotide resolution for every single transcript based on XGBoost (Extreme Gradient Boosting) model by directly using the raw signal around m^6^A sites (Fig. 1). We analyzed published Nanopore raw signals from modified and unmodified RRACH k-mers [10] and found that mean, median, standard deviation, and width of signals were different between modified or unmodified sites. Thus, we extracted the above features of all reads and divided them into training and testing datasets at a ratio of 4:1 to train an XGBoost model (Fig. 1). We then performed 10-fold cross-validation to evaluate the performance of the model. The area under the curve (AUC) was 97% (Additional file 1: Fig. S1a). We compared XGboost with several other classification algorithm, and the results showed that XGBoost presented the best performance. Thus, we used the XGBoost model in this study.

In order to validate that Nanom6A could identify m^6^A modification from individual transcripts, we evaluated our method using a known sample of synthesized mRNAs with known m^6^A to non-m^6^A ratios [10]. Firstly, we used the Nanom6A model to predict known modified and unmodified reads, respectively. In total, 91–96% of the modified and unmodified sites from individual transcripts could be successfully identified (Additional file 1: Fig. S1b-c). Secondly, we mixed known modified and unmodified reads together to simulate datasets with different m^6^A to non-m^6^A ratios, which covered the whole interval of modified ratio from 0 (all unmodified DRS reads) to 1 (all modified reads). The above simulated DRS dataset was analyzed by Nanom6A, which showed that the predicted m^6^A to non-m^6^A ratios based on Nanom6A presented a strong correlation with known m^6^A to non-m^6^A ratios (Additional file 1: Fig. S2). We also simulated different sequence depths to generate three different levels by mixing 20, 50, and 100 DRS reads in 10,000 times for each different m^6^A to non-m^6^A ratio, respectively. These results show that higher depth could generate a higher correlation (Additional file 1: Fig. S2).

### Validated Nanom6A using known m6A sites in both mammals and plants

**Fig. 2:**
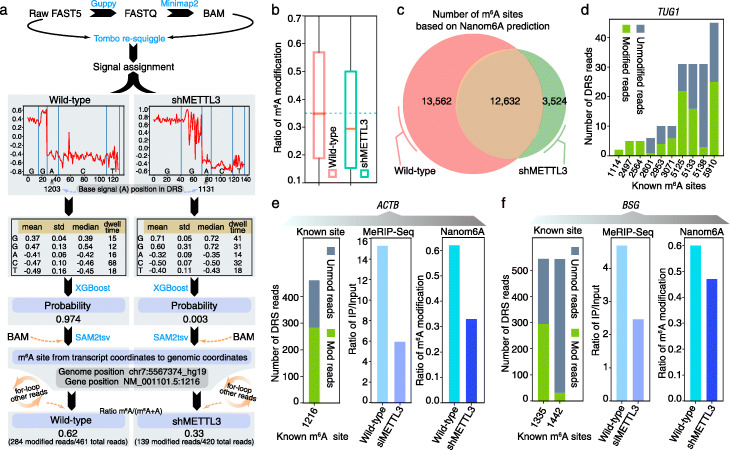
Validation of Nanom6A using known m^6^A sites in mammal. **a** Strategies to identify m^6^A site from ACTB site (1216) using ionic current signals in two DRS reads from wild-type and shMETTL3, respectively. **b** Bar plot shows the predicted m^6^A ratio from wild-type and shMETTL3, respectively. **c** Venn diagram of the number of predicted m^6^A sites from wild-type and shMETTL3, respectively. **d** Bar plot displays the consistency between known m^6^A sites based on the SCARLET method and Nanom6A prediction. The *x*-axis represents the modified sites in TUG1. The *y*-axis shows the number of modified reads. **e** Three bar plots show consistency between known modified sites in ACTB and Nanom6A prediction, enrichment of MeRIP-Seq IP over input reads, and Nanom6A-modified ratio, respectively. **f** Bar plots demonstrate consistency between known modified sites in BSG and Nanom6A prediction, enrichment of MeRIP-Seq IP over input reads, and Nanom6A-modified ratio, respectively

Before using this method in *Populus trichocarpa* (*P. trichocarpa*), we first used published DRS data in both mammals and plants to further validate the reliability of Nanom6A (Fig. 2a). Firstly, we used the raw signal file from both wild-type and METTL3 knockdown (shMETTL3) [11] to identify m^6^A sites using Nanom6A and compare with the known sites in ACTB site (1216) using ligation-assisted extraction and thin-layer chromatography (SCARLET) [15]. We extracted the mean, standard deviation, median, and dwell time after signal assignment using re-squiggle algorithm of Tombo (Fig. 2a). The modified probability in ACTB site (1216) was 0.974 and 0.003 for two DRS reads from wild-type and shMETTL3, respectively (Fig. 2a). The result was consistent with the reported modified site (1216) from ACTB using SCARLET [15]. Using the same strategies, we calculated the modified probability in ACTB site (1216) for other DRS reads. According to the formula of m^6^A reads/total DRS reads from ACTB, the m^6^A ratio of ACTB site (1216) was 0.62 and 0.33, respectively. We also used this method to calculate the ratio for all other genes, which were covered by DRS reads. The result showed that both modification ratio (Fig. 2b) and number of m^6^A sites (Fig. 2c) were decreased in the METTL3 knockdown sample.

To further evaluate the reliability of our method, we calculate all known m^6^A sites based on SCARLET [15]. In total, the m^6^A modification of two human lncRNAs (MALAT1 and TUG1) and three human mRNAs (TPT1, ACTB, and BSG) have been reported [15], which presented reliable known modification sites from transcripts in vivo. We compared the Nanom6A identified m^6^A sites based on DRS data [11] with known m^6^A modification based on the SCARLET method [15]. Among two lncRNAs (MALAT1 and TUG1), MALAT1 (NR_002819.2) was supported by only three DRS reads, which were too few to cover known m^6^A sites. Thus, we excluded the comparison of MALAT1 from downstream analysis. In TUG1 RNA, Nanom6A identified ten known modified sites, which included the site in 1114, 2601, 2497, 2564, 2953, 3071, 5125, 5133, 5138, and 5910 and were consistent with the SCARLET method (Fig. 2d). Especially, we found that these sites located near the 3′ terminal region included more DRS reads support. For TPT1 mRNA (NM_003295.1), Nanom6A-based DRS reads also presented consistent results with SCARLET in 687, 694, and 703 modified sites. ACTB mRNA (NM_001101.5) and BSG mRNA (NM_198591.1) have three known m^6^A modifications based on the SCARLET method [15]. Our method supported these modified sites and was consistent with SCARLET results (Fig. 2e, f). Notably, two modified sites showed a high modification ratio based on Nanom6A using DRS data at 1216 in ACTB and 1335 in BSG, respectively. Thus, we selected these m^6^A-modified sites for qualitative comparison between wild-type and METTL3 knockdown (shMETTL3). Using Nanom6A, the modification ratio at 1216 site from ACTB was 61.6% and 33.1% in wild-type and shMETTL3, respectively (Fig. 2e). The modification ratio at 1335 site from BSG transcripts was 60% and 47% in wild-type and shMETTL3, respectively (Fig. 2f). Published MeRIP-Seq dataset [16, 17] also reported a similar decreasing trend, which further validated the result based on Nanom6A.

**Fig. 3:**
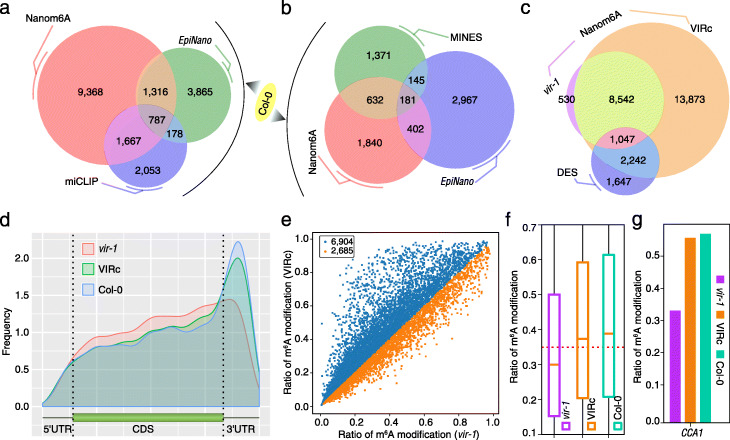
Validation of Nanom6A using known m^6^A sites in plant. **a** Comparison of all m^6^A sites among Nanom6A, miCLIP, and EpiNano. **b** Comparison of four motifs (AGACT, GGACA, GGACC, and GGACT sites) among Nanom6A, MINES, and EpiNano. **c** Comparison of m^6^A sites between reported differential error sites (DES) from a previous study and identified m^6^A based on Nanom6A from the *vir-1* mutant and *VIR*-complemented line, respectively. **d** The distribution of m^6^A sites from *vir-1*, VIRc, and Col-0, respectively. **e** Scatter plot showing the m^6^A ratio between *vir-1* and VIRc. **f** Box plot shows the m^6^A ratio in *vir-1*, VIRc, and Col-0. g Bar plot of *CCA1* shows m^6^A modification ratio from *vir-1*, VIRc, and Col-0, respectively

We further compared our method with several published methods on *Arabidopsis* DRS data [1] and revealed that 3770 m^6^A sites identified with the Nanom6A method overlapped with modification detected using *EpiNano* or miCLIP (Fig. 3a). Especially, Nanom6A reported 1667 m^6^A-modified sites detected with miCLIP, which were not detected by *EpiNano* (Fig. 3a)*.* For AGACT, GGACA, GGACC, and GGACT motifs, about 40% (1215/3055) m^6^A sites detected with the Nanom6A method overlapped with MINES or *EpiNano* (Fig. 3b)*.* We further compared 17,491 differential error sites (DES) from Parker et al. study [1], which included a total of 4936 sites with RRACH motif. About 66% (3289/6936) RRACH motifs from Parker et al. study [1] also reported by Nanom6A (Fig. 3c). The number of m^6^A sites based on Nanom6A using DRS data revealed an obvious decrease of m^6^A sites in *VIRILIZER* (*vir-1*) mutant as compared with *VIR*-complemented line (Fig. 3c). Interestingly, we found that the depletion of VIR reduces the enrichment of m^6^A sites in near the stop codon and 3′UTR (Fig. 3d). This result was consistent with the previously reported function of VIRMA, which mediates methylation in the stop codon and 3′UTR [18].

The ratio of m^6^A site modification (Fig. 3e, f) based on Nanom6A prediction was also decreased in *vir-1*, which is consistent with the function of VIR (a conserved m^6^A writer complex component) and further validated the reliability of our method. Interestingly, CCA1, a regulator of the circadian rhythm, has m^6^A modification in its mRNA in *Arabidopsis* [1]. Nanom6A also identified this modified site, which showed a decreased ratio of m^6^A site modification in the *vir-1* mutant (Fig. 3g).

### Qualitative profiling of m^6^A in stem-differentiating xylem of *Populus trichocarpa*

**Fig. 4:**
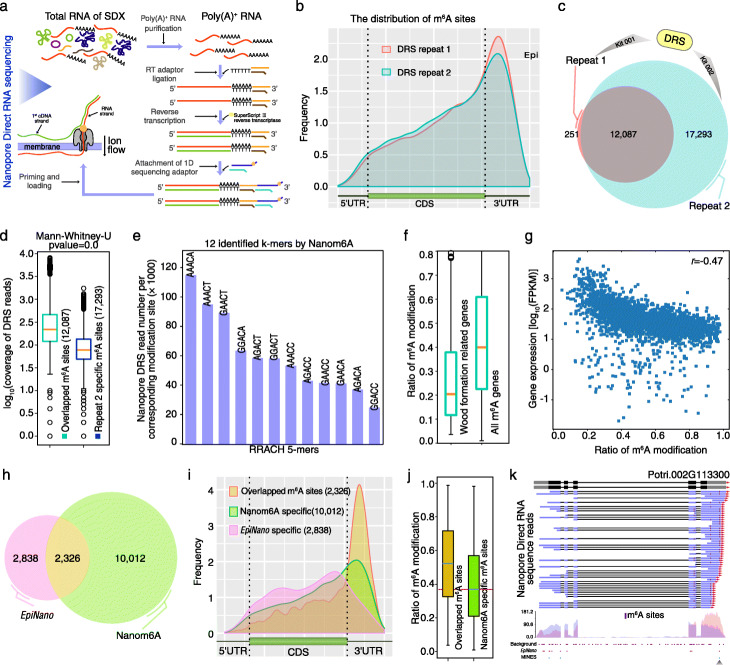
Quantitative profiling of m^6^A in stem-differentiating xylem of *Populus trichocarpa*. **a** Flowchart of DRS library construction and sequencing. **b** The distribution of m^6^A sites across the transcripts. **c** Comparison of m^6^A sites from two independent biological repeats. **d** Box plot shows DRS reads coverage of overlapped m^6^A sites and repeat2-specific m^6^A sites. **e** All the RRACH motif methylation supported by Nanopore DRS reads. **f** The m^6^A ratio for all the modified genes and wood formation-related genes. **g** The correlation between m^6^A ratio and gene expression. **h** Venn diagram shows the overlap between m^6^A sites predicted with Nanom6A and *Epinano*. **i** The distribution of m^6^A site for overlapped sites, Nanom6A-specific sites, and EpiNano-specific sites. **j** The m^6^A ratio for overlapped sites and Nanom6A-specific sites. **k** The modification sites of *ARK2* from DRS, *EpiNano*, MINES, and MeRIP-Seq. Blue and red colors in MeRIP-Seq represented Input and IP library, respectively

Using conserved domains of MTA70, WTAP, and YTH proteins that function in adenine methylation, demethylation, and readers of m^6^A, we identified all the major components (7 m^6^A methyltransferases, 15 m^6^A demethylases, and 18 m^6^A binding proteins), suggesting the conserved mechanisms of m^6^A modification in *P. trichocarpa.* We then extracted poly(A)^+^ RNA from stem-differentiating xylem (SDX) and performed direct RNA sequencing using both GridION and MinION platform (Fig. 4a). Firstly, the signals were correctly assigned to the corresponding bases before using the trained models (Fig. 1). After basecalling using Guppy (version 3.6.1), Nanopore long reads were mapped to the reference sequence (version 3) using minimap2 [19]. The re-squiggle module of Tombo (v1.5) was used to assign the raw signals to each individual base. Then, the raw signals from each individual sequence were extracted for m^6^A prediction using the trained XGBoost model (Fig. 1). In total, Nanopore DRS reads supported the expression of 22,953 genes. Among them, a total of 12,338 and 29,380 unique m^6^A sites were identified from repeat1 and repeat2, respectively. The average number of m^6^A sites for each transcript was 4. The distribution of m^6^A revealed that the modification is enriched near the stop codon and 3′UTR region (Fig. 4b), which is consistent with previous results [1] and further validated the reliability of our method*.*

Through two biological repeats using different sequence platform (GridION and MinION) and different kits (SQK-RNA001 and SQK-RNA002), Pearson’s correlation coefficient of 12,087 overlapped m^6^A sites was 0.96 (Additional file 1: Fig. S3), which revealed that Nanom6A provides good reproducibility between biological repeats even when we used different library preparation kits and different sequencers. In total, 98% m^6^A sites from the first repeat overlapped with the second repeat, suggesting high reproducibility between two independent biological repeats (Fig. 4c). Due to higher sequence depth, the second repeat identified 17,293 novel m^6^A sites, which were missed in the first repeat. Obviously, these repeat2-specific modified sites mainly originated from low abundance transcripts (Fig. 4d), which suggested that improving the depth could increase the chance to identify m^6^A sites. The average ratio of the m^6^A site was 0.44 in SDX. The distribution of supported DRS reads for each motif is presented in Fig. 4e. The lengths of exons containing m^6^A were longer than that of the control (Additional file 1: Fig. S4), in line with humans [9, 20].

Among the 76 genes associated with wood formation, transcripts from 17, 26, and 11 hemicellulose, lignin, and cellulose genes, respectively, contained m^6^A, suggesting that m^6^A modification is enriched in transcripts encoding proteins involved in wood formation. However, we found that the modification ratio of wood formation-related genes (~ 0.20) was lower than that of the average value (Fig. 4f). After investigating the top 1000 genes with the lowest m^6^A level, we found that genes involved in the lignin biosynthetic process (*P* = 5.54E^−15^), xylan biosynthetic process (*P* = 2.78E^−06^), and secondary cell wall biogenesis (*P* = 5.08E^−17^) were enriched. Furthermore, we found that gene expression levels and m^6^A levels showed a negative correlation (*r* = − 0.47) (Fig. 4g). Thus, it might be possible that the low m^6^A level in wood formation-associated genes in SDX is one of the factors that contributes to the stability of these transcripts and their high level of expression. In mammalian systems, it has been shown that m^6^A modification increases mRNA decay [21]. Further studies are needed to test if m^6^A modification regulates the level of expression of genes associated with wood formation.

Finally, we provided a comparison of our method with *EpiNano* (Fig. 4h–j) and MINES (Additional file 1: Fig. S5) using the same cutoff (> 20 DRS reads support). Venn diagram between Nanom6A vs. *EpiNano* depicted the overlap of 2326 m^6^A sites, 10,012 Nanom6A-specific and 2838 *EpiNano*-specific sites (Fig. 4h), respectively. The distribution of m^6^A sites showed that 2326 overlapped and 10,012 Nanom6A-specific m6A sites were more enriched near stop codon and 3′UTR region than that of 2838 *EpiNano*-specific sites (Fig. 4i). Especially, we found that the ratio of m^6^A from 10,012 Nanom6A-specific sites was lower than that of 2326 overlapped sites (Fig. 4j), which suggested that Nanom6A has an advantage in detecting low m^6^A/A ratio since Nanom6A could detect m^6^A at single transcript resolution. MINES only generated m^6^A sites for AGACT, GGACA, GGACC, and GGACT. Thus, we only compared the above four motifs using these three methods with MINES. The comparison between Nanom6A vs. MINES (Additional file 1: Fig. S5) also presented a similar trend with Nanom6A vs. *EpiNano*. Cellulose ARK2 included three m^6^A sites based on Nanom6A. The distal m^6^A site was identified by all methods (Fig. 4k).

### Validation of Nanom6A using MeRIP-Seq and m^6^A-REF-seq

**Fig. 5:**
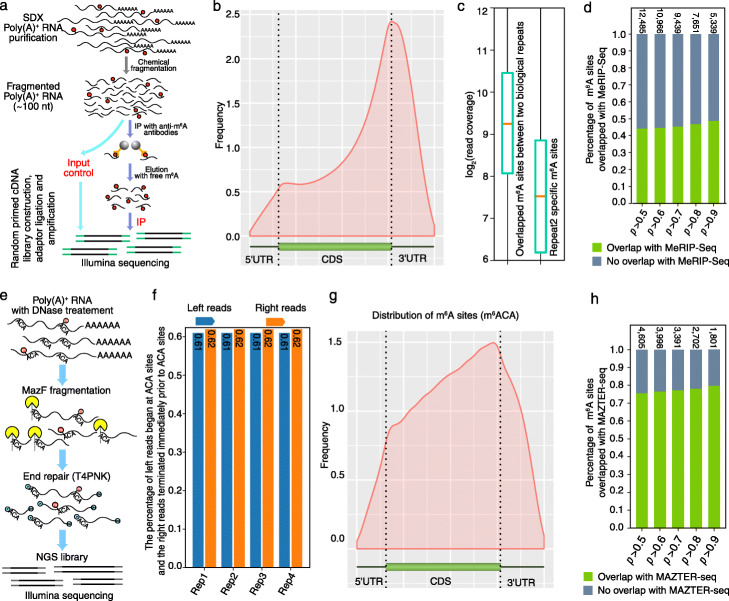
Validation of Nanom6A using MeRIP-Seq and m^6^A-REF-seq/MAZTER-seq. **a** Flowchart summarizing MeRIP-Seq. **b** The distribution of m^6^A in MeRIP-Seq reads across transcripts. **c** Box plot shows the reads coverage of overlapped m^6^A sites and repeat2-specific sites. **d** The percentage of m^6^A sites that were validated by MeRIP-Seq. **e** Flowchart of m^6^A-REF-seq/MAZTER-seq. **f** Bar plot shows the percentage of ACA motif at the end of sequence reads. **g** The distribution of ACA-modified sites across transcripts. **h** The percentage of m^6^A sites that were validated by m^6^A-REF-seq/MAZTER-seq

To further validate the predicted m^6^A sites based on Nanopore DRS, we also performed MeRIP-Seq with an anti-m^6^A antibody (Fig. 5a). The methylated RNA and the input RNA were subjected to the cDNA library construction using the KAPA Stranded mRNA-Seq Kit and sequenced on the Illumina Novaseq platform. Hisat2 (v2.0.3) [22] was used to align MeRIP-Seq reads to the genome and unique mapping reads were used for m^6^A peaks calling by PEA R packages (v1.1) [23]. The immunoprecipitated reads from MeRIP-Seq also showed the enrichment near stop codon and 3′UTR region (Fig. 5b). The overlapped m^6^A sites from two repeats presented higher coverage than that of novel m^6^A sites from the second repeat, which suggested higher sequencing depth for MeRIP-Seq could detect more m^6^A sites from low abundance transcripts (Fig. 5c). In total, 81% (2626/3253) of m^6^A-modified genes predicted by DRS were also detected by MeRIP-Seq. At single-base resolution, 49% of predicted m^6^A sites from the Nanopore DRS were covered by MeRIP-Seq peaks (Fig. 5d).

Recently, m^6^A-REF-seq [9] or MAZTER-seq [8] provide single-base resolution for m^6^A sites in ACA motif. To further validate the reliability of Nanom6A, we also performed m^6^A-REF-seq to validate the method based on direct RNA sequencing. We firstly perform m^6^A-REF-seq in *P. trichocarpa* following previous m^6^A-REF-seq library construction methods [9]. Then, m^6^A-REF-seq libraries were sequenced using the Illumina Nova6000 platform (Illumina, USA) (Fig. 5e). We found the enrichment of ACA motif at the end of sequencing reads and more than 60% left and right reads from Illumina pair-end model began and terminated at the ACA site, respectively (Fig. 5f). Our results on the percentage of ACA at the end of sequencing reads are consistent with a previous study [8], suggesting the high reliability of m^6^A-REF-seq. The m^6^A sites were identified using previously published scripts with default parameters [8]. As expected, the m^6^A sites in the ACA motif based on our m^6^A-REF-seq were enriched near the stop codon (Fig. 5g). In total, m^6^A-REF-seq validated 80% m^6^A sites based on Nanom6A (Fig. 5h).

### Poly(A) tail length in stem-differentiating xylem of *Populus trichocarpa*

**Fig. 6:**
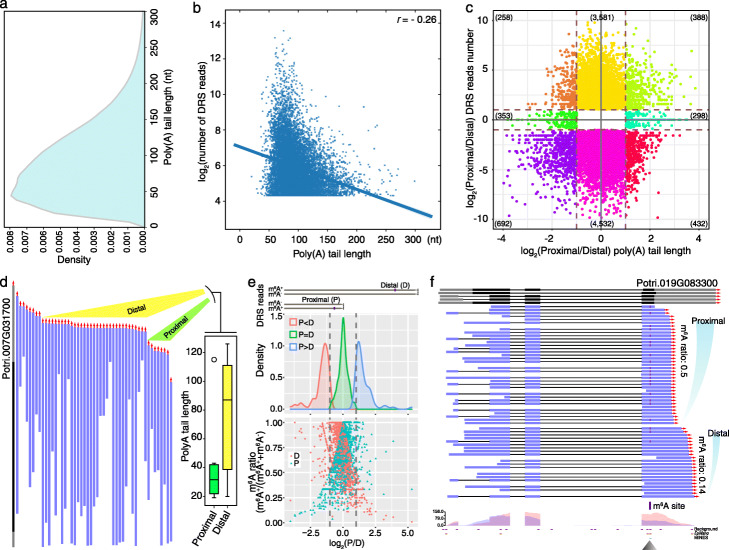
The differential length of poly(A) tail and differential m^6^A modification on alternative polyadenylation sites. **a** Density plot of poly(A) tail length based on nanopolish-polya. **b** Scatter plot showing the correlation between gene expression and poly(A) tail length. **c** Scatter plot shows the poly(A) tail length and DRS reads abundance from proximal and distal transcripts. **d** Gene structure and DRS reads of *PARVUS-L-2*, which has two polyadenylation sites with different poly(A) tail length. **e** Density plot in the upper panel shows a fold change of methylation ratio from distal (D) and proximal (P) poly(A) sites, respectively. The plot in the lower panel shows the m^6^A ratio from distal and proximal poly(A) sites, respectively. **f** Representative example for different m^6^A modifications for transcripts with distal or proximal poly(A) sites, respectively

Nanopore DRS has a great advantage in detecting poly(A) tail length, which has been reported in Arabidopsis [1], in vitro transcribed RNAs [24], and GM12878 cells [14]. In this study, we adopted a standard library preparation protocol for native RNA sequencing, which retained the full poly(A) tail to identify isoform-specific poly(A) tail length. We identified poly(A) tail length with a nanopolish-polya module [12] in the nanopolish package (0.11.1). We found that the mean and median poly(A) length for *P. trichocarpa* mRNA was 92 nt and 82 nt, respectively (Fig. 6a). The gene expression and poly(A) tail length showed a negative correlation (*r* = − 0.26) in *Populus* (Fig. 6b), which was consistent with the previous study [1]. In total, there were 2421 alternative polyadenylation genes included proximal and distal poly(A) sites, which showed two-fold change in poly(A) length (Fig. 6c). For example, PARVUS-L-2 is galacturonosyltransferase-like 1, which shows tissue-specific expression in stem-derived developing xylem [25]. Our DRS data showed that this secondary wall biosynthetic gene included two polyadenylation sites with different poly(A) length (Fig. 6d). The transcripts with distal polyadenylation presented longer poly(A) than that of proximal transcripts. This analysis provided preliminary poly(A) tail lengths from alternative polyadenylation to investigate the potential regulatory role of poly(A) tails in degradation and translation.

### Quantitative profiling of m^6^A in stem-differentiating xylem of *Populus trichocarpa*

The advantage of our method is that we can quantify m^6^A from raw Nanopore DRS data. Thus, the ratio of each m^6^A site was calculated based on the modified and unmodified transcripts since our method could detect m^6^A sites for each transcript at single-base resolution. Nanopore DRS could identify distal and proximal poly(A) sites. Poly(A) sites from DRS were grouped into a poly(A) site cluster when they were located within 24 nt of each other. A previous study has shown that an impaired m^6^A methylase complex can alter the usage of poly(A) sites [6]. Among all m^6^A-modified genes, we found 3152 genes using alternative polyadenylation (APA). Moreover, our method in this study could distinguish m^6^A and non-m^6^A transcripts. The distribution of methylation ratio revealed that the transcripts with distal and proximal poly(A) sites have different percentages of modification based on methylated and nonmethylated DRS transcripts (Fig. 6e). For example, transcripts with distal poly(A) from Potri.019G083300 have fewer m^6^A sites than that of proximal poly(A) transcripts (Fig. 6f). This study provided preliminary data to further investigate the m^6^A-modified isoforms coupled with alternative polyadenylation.

## Conclusions

Recently, the Nanopore direct RNA sequencing is used to detect base modification signals in RNA [1], which has been reported by both *EpiNano* [10] and MINES [11]. However, *EpiNano* [10] could not distinguish m^6^A from other types of RNA modification, such as m^1^A as this method was based on base quality and deletion frequency to predict m^6^A sites. MINES [11] detects m^6^A sites only in certain sequence contexts (AGACT, GGACA, GGACC, and GGACT). In this study, we developed Nanom6A, a new pipeline for the identification of m^6^A modification at a single-base resolution to overcome the limitations with the present methods using Nanopore direct RNA reads based on our XGBoost model, which is distinct from previous methods that used SVM [10] or random forest [11]. Importantly, unlike published methods for DRS data, the abundance of m^6^A sites could be quantified by our Nanom6A pipeline since this method could provide m^6^A modification at transcript resolution. To validate our method based on Nanopore RNA direct sequencing, we used MeRIP-Seq and m^6^A-REF-seq [9], which revealed that Nanom6A could achieve high accuracy in detecting m^6^A for both qualitative and quantitative analysis. Using this method, we provided a transcriptome-wide identification and quantification of m^6^A modification in SDX at transcript-based resolution and revealed that different APA usage showed a different ratio of m^6^A. Our method largely expands the application of Nanopore direct RNA sequencing in exploring the regulatory mechanisms of m^6^A.

## Methods

### Plant material

*P. trichocarpa* were grown in soil consisting of natural soil and peat moss (PINDSTRUP PLUS) in the ratio 2:1 in a greenhouse under the average temperature of 22 °C and light/dark cycle of 16/8 h. Plants were watered every 2 days and fertilized once a month using N/P/K compound fertilizer [26]. Stem below the seventh leaf was debarked and scraped as SDX. The SDX materials were wrapped with tinfoil, then dropped into liquid nitrogen immediately. Finally, the materials were stored at the ultra-low temperature freezer at − 80 °C for downstream Nanopore direct RNA sequencing, MeRIP-Seq and m^6^A-REF-seq.

### Total RNA extraction and quality assessment

Total RNA was extracted using RNAprep Pure Plant Kit (polysaccharides and polyphenolics-rich) (Tiangen, no. DP441, China) and treated with DNase I to remove DNA. Firstly, the quality of total RNAs was detected using 1% agarose electrophoresis and did not observe any RNA degradation. Then, the OD260/280 for all the samples was at 2.12–2.15 using a Nanodrop 2000 spectrophotometer (Thermo Scientific). Finally, the RIN value for all the samples was above 9.0 using the Agilent 2100 Bioanalyzer in combination with RNA Analysis Kits.

### Construction of library and direct RNA sequencing

Libraries for direct RNA sequencing were prepared following the ONT Direct RNA Sequencing protocol with minor modification. Enrichment of poly(A)^+^ RNA was performed using mRNA Dynabeads™ mRNA purification kit (Thermo, 61006). The Nanopore RTA adapter was ligated to the 300 ng poly(A)^+^ RNA using T4 DNA ligase (NEB-M0202M) at 25 °C for 20 min and was reverse transcribed using SuperScript III reverse transcriptase (Thermo Fisher, 18080093) to form RNA/DNA duplexes and relax the secondary structure of RNA. The products were purified using 1.8 × (72 μl) VAHTS RNA Clean Beads (Vazyme-N412-01-AA), washed with 70% freshly prepared ethanol, then eluted using 21-μl nuclease-free water (Promega, USA) (1-μl eluted product was used to quantify the concentration using QubitTM dsDNA HS Assay Kit). The RNA adapter (RMX) was ligated onto the 3′ end of RNA:DNA hybrid with RTA at 25 °C for 40 min, and the mix was purified using 1X VAHTS RNA Clean Beads, washed two times with washing buffer (WSB). The products were then eluted in 21-μl Elution Buffer (1-μl elution was used to quantify the concentration using QubitTM dsDNA HS Assay Kit), mixed with nuclease-free water and RNA Running Buffer (RRB) to a total of 75-μl library prior to loading onto the flow cell, and ran on GridION (repeat1) and MinION (repeat2) sequencer, respectively. The RNA strand was ligated to the 1D sequencing linker bearing the RNA motor protein, which ensured efficient translocation of RNA rather than cDNA through the Nanopore [27, 28]. Two independent biological repeats were sequenced using a GridION X5 sequencer with flow cell R9.4.1 and a MinION flow cell (FLO-MIN106) to generate real-time single-molecule sequencing.

### Feature extraction and training using XGBoost classifier

In this study, we used a distinct algorithm to identify m^6^A from raw signal directly. Firstly, the Nanopore raw signals from in vitro transcript of the modified and unmodified sequence were downloaded from *EpiNano* [10]. MINES [11] corrected the raw signal through Tombo re-squiggle function. In this study, we also used this method to correct the raw basecalling sequence and assign the corrected base to the raw signal segment [29]. We searched for RRACH motif and extracted the median, standard deviation, mean, and number of Nanopore signals from each RRACH motif. Then, we divided the extracted features into training datasets and testing datasets with the ratio of 4:1. Extreme Gradient Boosting (XGBoost) is an ensemble algorithm of decision trees and has been widely used in all kinds of data mining fields [30]. It was an ensemble method based on a gradient boosted tree (gbtree). In the regression trees, the inside nodes represent values for an attribute test and the leaf nodes with scores represent a decision; the sum of the scores is predicted by K trees as below:

$$ {\hat{\mathrm{y}}}_i={\sum}_{k=1}^K{f}_k\left({x}_i\right),\kern0.5em {f}_k\kern0.5em \in \kern0.5em F $$

$$ obj\left(\theta \right)={\sum}_{i=1}^n\mathrm{loss}\left({y}_i,{\hat{y}}_i\right)+{\sum}_{i=1}^K\Omega \left({f}_k\right) $$

*f*_*k*_(*xi*) is the score of the *k*th tree, *F* is the space function of all regression trees, the loss function measures whether the model is suitable for training set data, and the Ω function is used for punishing the model complexity [31]. The XGBoost model was constructed using the machine learning library Scikit-learn [32].

We used the following three parameters for evaluation:

$$ \mathrm{TPR}=\frac{\mathrm{TP}}{\mathrm{TP}+\mathrm{FN}}\kern0.5em \mathrm{FP}\mathrm{R}=\frac{\mathrm{TN}}{\mathrm{FP}+\mathrm{TN}}\kern0.5em \mathrm{Accuracy}=\frac{\mathrm{TP}\kern0.5em +\kern0.5em \mathrm{TN}}{\mathrm{TP}\kern0.5em +\kern0.5em \mathrm{FP}\kern0.5em +\kern0.5em \mathrm{TN}\kern0.5em +\kern0.5em \mathrm{FN}} $$

TP, FP, TN, and FN represented true positives, false positives, true negatives, and false negatives, respectively. The receiver operating characteristic (ROC) curve and the area under the curve (AUC) were used to measure a predictive power [33]. In this study, the input dataset was raw signals from Nanopore direct RNA sequencing. The output result was the prediction class and probability.

### Identification of modified m^6^A sites from SDX

After basecalling using Guppy (version 3.6.1), Nanopore reads from direct sequencing of SDX were aligned to *P. trichocarpa* genome (v3.0) using minimap2 [19] with --secondary=no -ax splice -uf -k14 option. We performed re-squiggle raw current signal with tombo (v1.5). Firstly, the electric current signal from raw reads (in FAST5 format) and associated base calls is assigned to transcriptome reference based on the expected current level model using the re-squiggle algorithm from Tombo. In brief, basecalled reads located within the FAST5 file were mapped to transcriptome reference using python API of mimimap2. The raw signal from direct RNA sequencing reads was normalized using the median shift and median absolute deviation scale parameters. The segmented signal was determined by identifying a large shift in the current level. The most likely matching between transcript sequence and signal was determined using the signal assignment algorithm in Tombo. Secondly, we extracted the median, standard deviation, mean, and dwell time from Nanopore raw signal data in each direct RNA sequencing read to calculate the probability of m^6^A modification after the signal to transcript sequence assignment. Thirdly, SAM2Tsv [34] generated TAB-delimited m^6^A site information including transcript coordinates and genomic coordinates. Finally, modified A base supported by at least 20 modified transcripts were identified as modified m^6^A sites. Nanom6A was developed to train, identify, and visualize the m^6^A of DRS reads, which is available at https://github.com/gaoyubang/nanom6A.

### Library construction and bioinformatics analysis for MeRIP-Seq

The MeRIP-Seq (m^6^A-seq) was performed as previously described with minor modifications [35]. Poly(A)^+^ RNA was purified from 300 μg total RNA using a Dynabeads™ mRNA purification kit (Thermo, 61006) and was randomly sheared into approximately 100-nt fragments by incubation for 5.5 min at 70 °C with 20-μl reaction system including 18-μl RNAs (about 5~6 μg of RNAs) and 2-μl 10 × Fragmentation Buffer (Ambion, AM8740). For purification of fragmented mRNAs, we mixed 1/10 volumes of 3 M sodium acetate (pH 5.2), 0.8-μl glycogen (15 mg/ml), and 2.5 volumes of 100% pre-cold ethanol, then incubated the mixture at − 80 °C overnight. We centrifuged the tube at 12,000 rpm at 4 °C for 25 min to pellet mRNAs, washed the mRNA pellets with 1-ml pre-cold 75% ethanol once, and centrifuged at 12,000 rpm for 15 min at 4 °C. Then, we air-dried the mRNA pellets in 15~20 min and dissolved in 200-μl nuclease-free water. About 50-ng fragmented mRNAs were used as input control. In total, 5-μg fragmented mRNAs was diluted to 766 μl with nuclease-free water, then mixed with 10-μl RiboLock RNase Inhibitor (40 U/μl; Thermo, E00381), 200-μl 5 × IP Buffer [50 mM Tris-HCl (pH 7.4), 750 mM NaCl, and 0.5% (v/v) Igepal CA-630], and 24-μl anti-m6A polyclonal antibody (0.42 mg/ml; Synaptic Systems, 202003). The 1-ml IP reaction was incubated at 4 °C for 2 h with ~ 10 rpm rotation. Meanwhile, we washed 50-μl Magna ChIP™ Protein A+G Magnetic Beads (Millipore, 16-663) twice with 500-ml 1 × IP Buffer (supplemented with RiboLock RNase Inhibitor) for each time. We discarded the supernatant from the beads and transferred the 1-ml IP mixture to the beads to incubate at 4 °C for another 2 h with rotation. Beads were spun down and the supernatant carefully removed and the beads with 1-ml 1 × IP Buffer (supplemented with RiboLock RNase Inhibitor) washed four times. The bound mRNAs were eluted with 100-μl Elution Buffer [10 mM Tris-HCl (pH 7.4), 150 mM NaCl, 0.1% (v/v) Igepal CA0630, 7-μl RiboLock RNase Inhibitor, and 6.7 mM m^6^A (Sigma, M2780)] at 4 °C for 1 h in Metal Bath (Eppendorf) with vigorous shaking (at least 1200 rpm). We repeated elution two times and combine the three elutes (300 μl in total), then added 1/10 volume (30 μl) of 3 M sodium acetate (pH 5.2) and 2.5 volumes (750 μl) of 100% ethanol to the eluate, and precipitated at − 80 °C overnight. RNA was pelleted by centrifugation and washed two times with 1-ml pre-cold 75% cold ethanol. The RNA was air-dried and dissolved in 10-μl nuclease-free water. The ~ 50 ng of immunoprecipitated mRNAs (IP) and pre-immunoprecipitated mRNAs (Input) were subjected to the cDNA library construction by using the KAPA Stranded mRNA-Seq Kit Illumina® platform and paired-end sequencing performed on the Illumina Novaseq platform (Illumina Inc., San Diego, CA, USA).

Raw data from MeRIP-Seq was aligned to the reference genome using Hisat2 (v2.0.3) [22] with --qc-filter option. Duplication reads were removed using samtools rmdup (v1.3.1). Finally, m^6^A peaks were called using PEA R package with exomePeak methods [23, 36] using the default option. Gene Ontology (GO) enrichment analysis was performed using clusterprofile [37].

### Library construction and bioinformatics analysis for m^6^A-REF-seq

Libraries for m^6^A-REF-seq were constructed using published methods [9] with minor changes. In brief, 1-μg Poly(A) + RNA was enriched by two-round purification from total RNA using DynabeadsTM mRNA Purification Kit (Thermo Scientific, 61006). We divided enriched poly(A) + RNA into several 200-ng reaction systems. Firstly, ~ 200 ng poly(A) + RNAs were first heated at 80 °C for 2 min and immediately placed on ice for at least 2 min. Then, the digestion reaction of MazF was conducted with 4-μl 5× MazF Buffer, 0.5-μl RNase inhibitor (TaKaRa, 2313A), 40 units of MazF enzyme (TaKaRa, 2415A), and nuclease-free water (Promega, USA) in 20-μl reaction mixture system. The digestion reaction was incubated at 37 °C for 30 min and stopped by placing it on ice. Then, fragmented RNAs were pooled together and purified by an RNA Clean & Concentrator Kit (TIANMO BIOTECH, TR115-50, China) and eluted in 25-μl nuclease-free water (70 °C). The concentration of the RNA fragment was measured by the Qubit RNA HS Assay Kit (Thermo Scientific, Q32852). The fragments after MazF digestion were visualized in 1.5% agarose electrophoresis. Finally, the fragmented RNAs were end-repaired using the T4 Polynucleotide Kinase (T4PNK, NEB, M0201) and incubated at 37 °C for 40 min in 50-μl reaction system, which was supplemented with 5-μl T4PNK Reaction Buffer, 5-μl ATP (NEB, B0756A), and 0.5-μl RNase inhibitor. The end-repaired fragments were purified with RNA Clean & Concentrator Kit (TIANMO) and the concentration evaluated using Qubit (Thermo Scientific) before library construction. The NGS libraries were constructed using the NEBNext® Multiplex Small RNA Library Prep Set for Illumina with 100 ng of end-repaired fragmented RNA using the following adapter sequence: 5′-AATGATACGGCGACCACCGAGATCTACACGTTCAGAGTTCTACAGTCCGACGATC (insert)AGATCGGAAGAGCACACGTCTGAACTCCAGTCACIIIIIIATCTCGTATGCCGTCTTCTGCTTG-3′. Finally, amplified libraries with 16 cycles for PCR enrichment were cleaned with 0.8× CleanNGS DNA & RNA Cleanup For NGS (CleanNA, CNGS-0500) and further quantified using Qubit (Thermo Scientific). Size selection was visualized in 1.5% agarose gel. Libraries were sequenced with 150-nt paired-end sequencing on an Illumina Novaseq 6000 platform.

The adapters of paired-end reads were filtered by cutadapt [38]. Reads were aligned to the reference genome using HISAT2 with default parameters [22]. The candidate ACA site and cleavage efficiency were calculated using MAZTER-mine R package with default options [8].

### Measurement of poly(A) tail length

We identified poly(A) tail length with nanopolish-polya module [12] in nanopolish package (0.11.1) (https://github.com/adbailey4/nanopolish/tree/cigar_output) with default options and qc_tags with “PASS” value were kept for downstream analysis.

#### Review history

The review history is available as Additional file 3.

#### Peer review information

Andrew Cosgrove was the primary editor of this article and managed its editorial process and peer review in collaboration with the rest of the editorial team.

## Supplementary Information

**Additional file 1.** Supplemental figures.

**Additional file 2.** List of datasets used.

**Additional file 3.** Review history.

## Data Availability

All raw signals from Nanopore direct RNA sequencing for two independent biological repeats were deposited in the SRA under the SRA accession SRR8491764 [39] (GridION sequencer) and SRR12676675 [40] (MinION sequencer), respectively. All the data from MeRIP-seq and m^6^A-REF-seq have been deposited in NCBI under accession PRJNA601096 [41] and PRJNA667190 [42], respectively. A comprehensive list of SRA accessions including Nanopore direct RNA sequencing and MeRIP-seq used in this study is available in Additional file 2.
