# Supplementary material for: Quantitative profiling of N6-methyladenosine at single-base resolution in stem-differentiating xylem of Populus trichocarpa using Nanopore direct RNA sequencing
Source: Genome Biol. 2021 Jan 7;22:22. doi: 10.1186/s13059-020-02241-7 (PMC7791831; doi:10.1186/s13059-020-02241-7)
Supplement: Supplementary file 1 — Additional file 1. Supplemental figures. [file 13059_2020_2241_MOESM1_ESM.pdf]

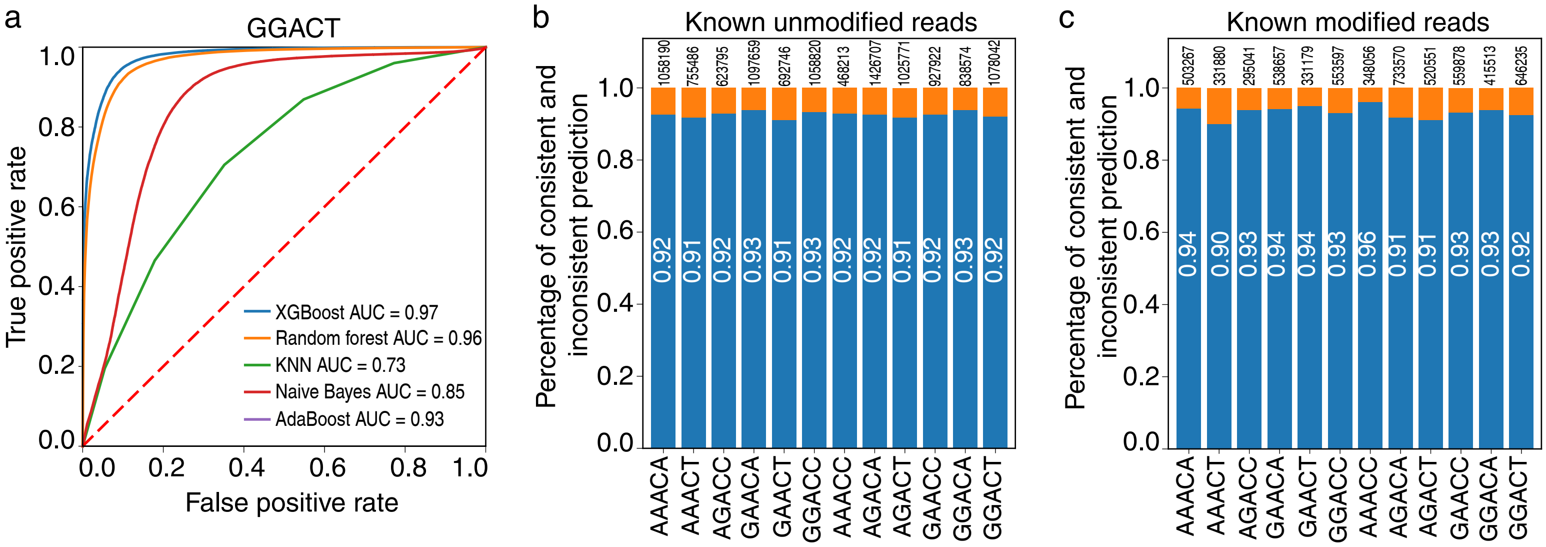

Figure S1: (a) ROC curve from several models (using one of the k-mer GGACT as an example). (b) Bar plot of predicted accuracy for all unmodified RRACH k-mers. (c) Bar plot of predicted accuracy for all modified RRACH k-mers.

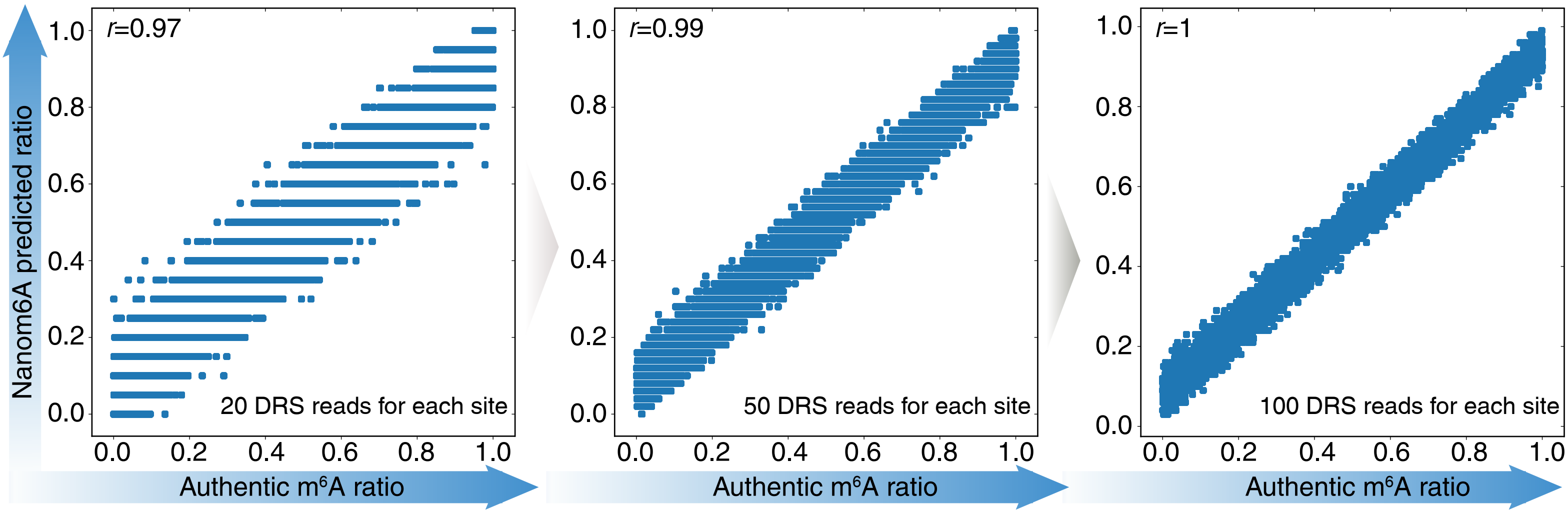

Figure S2: Scatter plot shows the correlation between authentic m^6A ratio and Nanom6A prediction. The x-axis represents known m^6A ratio (authentic m^6A-modified reads/authentic unmodified reads) based on random sub-sample of synthetic DRS transcripts. The y-axis represents the predicted m^6A ratio based on Nanom6A. The number of random rounds is 1000. Three panels from left to right represent 20, 50, and 100 DRS reads for each m^6A ratio, respectively.

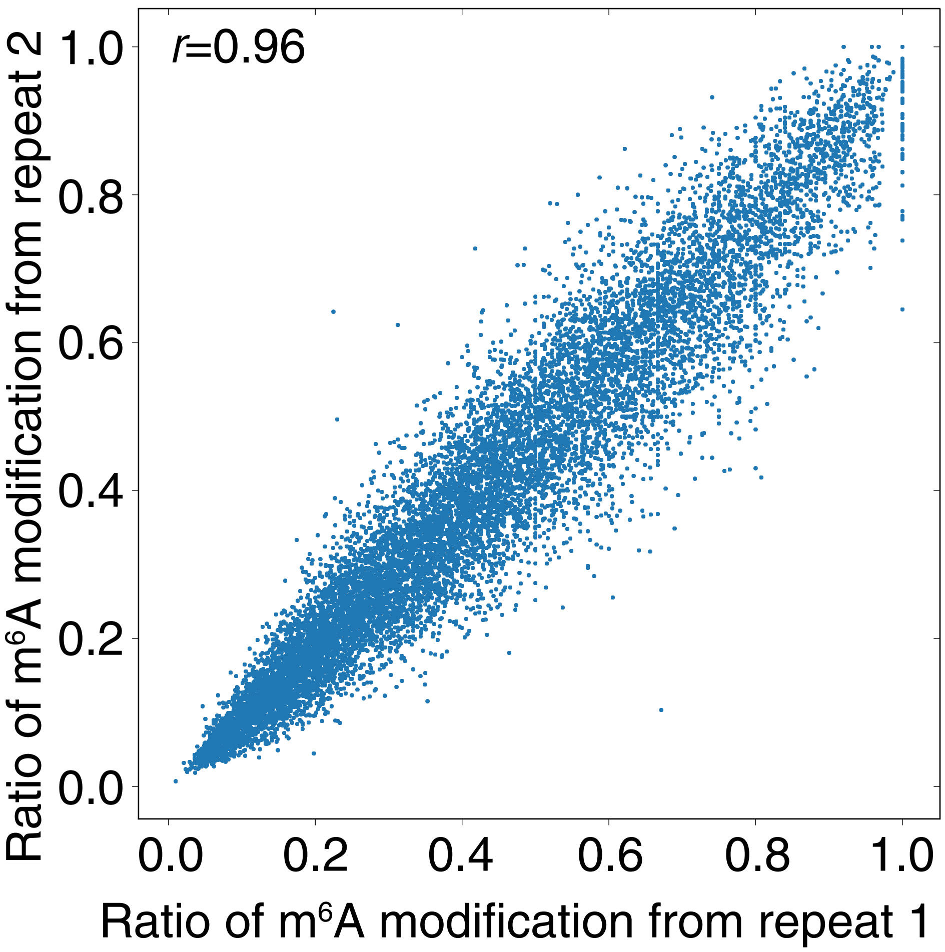

Figure S3: The correlation between two biological repeats.

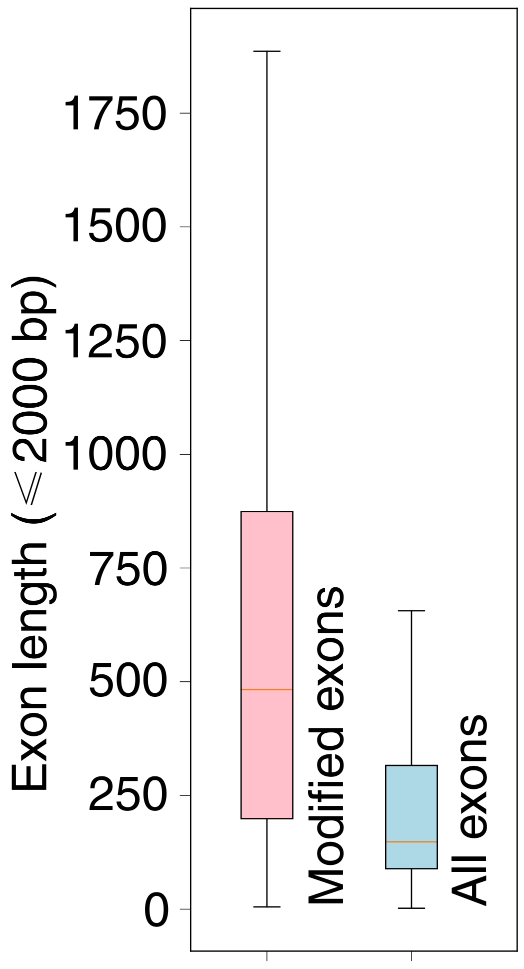

Figure S4: The length of exons with or without m^6A modification.

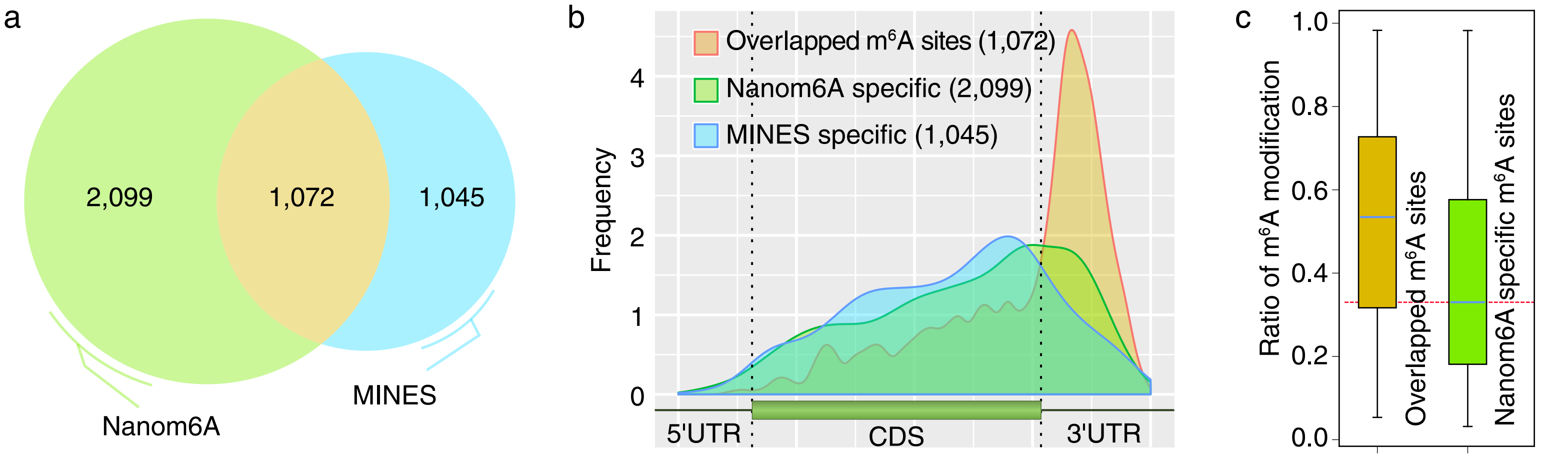

Figure S5: (a) Comparison of m^{6}A sites from AGACT, GGACA, GGACC, and GGACT sites between Nanom6A and MINES. (b) The distribution of m^{6}A site for overlapped sites, Nanom6A specific sites and MINES specific sites. (c) The m^{6}A ratio for overlapped sites and Nanom6A specific sites.
