# Supplementary material for: Quantitative profiling of N6-methyladenosine at single-base resolution in stem-differentiating xylem of Populus trichocarpa using Nanopore direct RNA sequencing
Source: Genome Biol. 2021 Jan 7;22:22. doi: 10.1186/s13059-020-02241-7 (PMC7791831; doi:10.1186/s13059-020-02241-7)
Supplement: Supplementary file 3 — Additional file 3. Review history. [file 13059_2020_2241_MOESM3_ESM.docx]

Review History

**First round of review**

**Reviewer 1**

**Were you able to assess all statistics in the manuscript, including the appropriateness of statistical tests used?**

No, I do not feel adequately qualified to assess the statistics.

**Comments to author:**

This manuscript definitely attempts to address an important problem in the fields of nanopore sequencing and RNA modification biology. We currently do not have a way to sequence RNA modifications at base resolution and obtain the exact modification fraction at each modified site. This is what this manuscript claimed to have achieved. My view of the work is that there isn't enough information for me to be convinced:

1. I did not see how the authors can assign modification fraction based on peak features. There need to be careful description, using known m6A sites on rRNA or mRNA in which we know modification fractions already to validate.

2. There has been two papers reporting the same strategy of applying MazF to sequence a small set of m6A sites in m6ACA context. Estimated modification fractions were obtained. The authors need to at least validate their method based on the known results. Tao Pan lab has reported modification fraction of a few sites using SCARLET. Those sites need to be validated.

3. More experimental and computational detailed are required in order to analyze the claims by the authors.

**Reviewer 2**

**Were you able to assess all statistics in the manuscript, including the appropriateness of statistical tests used?**

Yes, and I have assessed the statistics in my report.

**Comments to author:**

The manuscript by Gao and Liu et al (2020) applied an XGBoost model to identify m6A modified bases (Nanom6A) in direct RNA nanopore sequencing (dRNS) data. The authors compared their XGBoost model against two previously published methods, EpiNano [1] and MINES [2], using previously published Arabidopsis dRNAS data [3]. The study claims that the number of m6A sites identified by Nanom6A is comparable to MINES. Further, the authors claim that Nanom6A pipeline is superior because it uses the raw ionic current information that is extracted using Tombo. I liked that the authors looked at the distribution of m6A across transcripts and on the poly(A) tail. These are worthy endeavors especially for studying plant biology.

Major comments:

The department of Biology and Program in Cell and Molecular Biology, Colorado State University, Fort Collins, CO, United States is not attributed to any author.

Lines 56-57: "other known RRACH (D=G/A, H=A/U/C) motif" Should the parentheses contain R=G/A?

The principle of using ionic current signals to detect modified RNA nucleotides has been described in several studies, including Smith et al [4], Workman et al [5], Ding et al [6], Lorenz et al [2]. Additionally, MINES [2] uses the same Tombo resquiggle function that is used by the authors in this study. These claims should be toned down, and appropriate citations should be included.

Was a single Nanopore sequencing experiment sufficient to capture the transcriptome? Would the robustness of the data improve with replicates? And more sequencing should improve depth for low abundance transcripts?

Albacore basecaller has been deprecated by ONT for over a year. Why did the authors choose it in place of Guppy which has superior performance? The data analyses and inferences would substantially improve with re-basecalling using Guppy RNA flip-flop model.

The authors did not compare Nanom6A against the 17,491 m6A sites from the Parker et al study [3]. This is an important comparison to be performed since a lot of Nanom6A development used those data.

Also, the comparisons with other methods (EpiNano and MINES) needs to be performed one at a time (Nanom6A vs. EpiNano for all motifs, and Nanom6A vs. MINES for the four motifs). The claim that Nanom6A can identify m6A in context independent motifs needs to be substantiated.

The authors claim that Nanom6A identified 27% of m6A sites uniquely in comparison with EpiNano and MINES. Using the numbers from Figure 2c, a total of 55,213 m6A sites were identified. There were only 2,317 that were only identified by Nanom6A. That's only 4.1%. If we eliminate the EpiNano counts, there are 13,389 m6A sites identified by MINES and Nanom6A. Those 2,317 Nanom6A sites now account for ~17% of the total, still not 27%. How are they calculating the percentage of Nanom6A only sites? This needs clarity and a better explanation.

Why is this three-way comparison of techniques not established before applying Nanom6A to Populus trichocarpa SDX. It is important to demonstrate and compare Nanom6A on Arabidopsis data, followed by applying it on the dRNS data collected for P. trichocarpa.

The authors performed one MeRIP-seq experiment. Are replicates needed/useful?

Would higher sequencing depth for MeRIP-seq resolve issues with low abundance transcripts?

Figure 2 is busy and needs simplification. For example, panels 2d and 2e could be moved to the supplementary section. Each panel needs proper explanation. E.g. 2e computes log for number of reads. This really needs to be the raw counts (or raw counts + normalized proportions). 2b and 2c can be a table.

Line 138: What r value is being calculated for MeRIP-seq and Nanom6A? An r value of 0.53 seems low for trying to precisely predict m6A sites in P. trichocarpa.

Line 157: Is there a poly(A) tail length difference between reads using a canonical poly(A) site and an alternative poly(A) site?

Line 178: I'm not convinced that the authors have achieved m6A identification of individual transcripts without evaluating a sample with known m6A to non-m6A ratios. At minimum, the authors need to perform and include control experiments, and tone down the claims about quantification.

Line 204: Which reverse transcriptase was used for first strand synthesis? Did the authors follow standard protocol recommended by ONT?

The motivation for using the XGBoost model was never described by the authors. I appreciate it is an ensemble method, but it is important to explain why it could be applied to Nanopore ionic current data.

The Nanom6A pipeline code is not open source. The authors provide a Docker container but not the code itself. I can understand if there is a reason for this, but it was not provided in the manuscript.

Also, the SRR8491764 accession is not public so I could not download the data.

Minor comments:

The manuscript needs editing. There are problems with grammar and word use.

Some examples are below

Line 25 - omit "conservative" replace with "conserved"

Line 27 - Replace in single-base with "at the single base level"

Line 30 - add the word "an" before XGBoost model

Line 38 - replace "in" with "for"

Line 65 - omit distinct, insert "distinguish"

Line 254 - The remaining 1/10... (sentence doesn't make sense)

Figure 1 - spelling separate not seperate

Overall, the authors present an interesting manuscript that could be improved.

References:

1. Liu H, Begik O, Lucas MC, Ramirez JM, Mason CE, Wiener D, et al. Accurate detection of mA RNA modifications in native RNA sequences. Nat Commun. 2019;10:4079.

2. Lorenz DA, Sathe S, Einstein JM, Yeo GW. Direct RNA sequencing enables m6A detection in endogenous transcript isoforms at base-specific resolution [Internet]. RNA. 2020. p. 19-28. Available from: http://dx.doi.org/10.1261/rna.072785.119

3. Parker MT, Knop K, Sherwood AV, Schurch NJ, Mackinnon K, Gould PD, et al. Nanopore direct RNA sequencing maps the complexity of Arabidopsis mRNA processing and mA modification. Elife [Internet]. 2020;9. Available from: http://dx.doi.org/10.7554/eLife.49658

4. Smith AM, Jain M, Mulroney L, Garalde DR, Akeson M. Reading canonical and modified nucleobases in 16S ribosomal RNA using nanopore native RNA sequencing. PLoS One. 2019;14:e0216709.

5. Workman RE, Tang AD, Tang PS, Jain M, Tyson JR, Razaghi R, et al. Nanopore native RNA sequencing of a human poly(A) transcriptome. Nat Methods. 2019;16:1297-305.

6. Ding H, Bailey AD, Jain M, Olsen H, Paten B. Gaussian Mixture Model-Based Unsupervised Nucleotide Modification Number Detection Using Nanopore Sequencing Readouts. Bioinformatics [Internet]. 2020; Available from: http://dx.doi.org/10.1093/bioinformatics/btaa601

**Authors Response**

**Point-by-point responses to the reviewers’ comments:**

Response to Reviewers’ Reports

Reviewer #1: This manuscript definitely attempts to address an important problem in the fields of nanopore sequencing and RNA modification biology. We currently do not have a way to sequence RNA modifications at base resolution and obtain the exact modification fraction at each modified site. This is what this manuscript claimed to have achieved. My view of the work is that there isn't enough information for me to be convinced:

*Response: We appreciate your comments on the importance of this work, Thank you for your constructive comments and suggestions on our work. In the revised manuscript, we addressed your concerns by performing suggested experiments and data analysis. Below is our point-by-point response to the concerns you raised.*

1. I did not see how the authors can assign modification fraction based on peak features. There need to be careful description, using known m6A sites on rRNA or mRNA in which we know modification fractions already to validate.

*Response: We are sorry that we did not describe the assignment of modification fraction more clearly in the previous version of our manuscript. As suggested, we have added the description using known m6A site from mRNA of ACTB using site-specific cleavage and radioactive-labeling followed by ligation-assisted extraction and thin-layer chromatography (SCARLET) (Liu et al., 2013), which can quantify individual m6A sites. The following sections provide a detailed explanation and illustration.*

*Firstly, we used the raw signal file from both wild-type and METTL3 knockdown (shMETTL3) (Lorenz et al., 2020) to identify m6A sites using Nanom6A and compare with the known sites in ACTB site (1216) using ligation-assisted extraction and thin-layer chromatography (SCARLET) (Liu et al., 2013). We extracted the mean, standard deviation, median and dwell time after signal assignment using re-squiggle algorithm of Tombo (Fig. 2a). The modified probability in ACTB site (1216) was 0.974 and 0.003 for two DRS reads from wild-type and shMETTL3, respectively (Fig. 2a). The result was consistent with the reported modified site (1216) from ACTB using SCARLET (Liu et al., 2013). We have added Figure 2a and updated the text accordingly in the revised manuscript.*

*We also added more careful description in Method section: We performed re-squiggle raw current signal with tombo (v1.5). Firstly, the electric current signal from raw reads (in FAST5 format) and associated base calls is assigned to transcriptome reference based on the expected current level model using the re-squiggle algorithm from Tombo. In brief, basecalled reads located within the FAST5 file were mapped to transcriptome reference using python API of mimimap2. The raw signal from direct RNA sequencing reads was normalized using the median shift and median absolute deviation scale parameters. The segmented signal was determined by identifying a large shift in the current level. The most likely matching between transcript sequence and signal was determined using the signal assignment algorithm in Tombo. Secondly, we extracted the median, standard deviation, mean, and dwell time from Nanopore raw signal data in each direct RNA sequencing read to calculate the probability of m6A modification after the signal to transcript sequence assignment. Thirdly, SAM2Tsv (Lindenbaum, 2015) generated TAB delimited m6A sites information including transcript coordinates and genomic coordinates. Finally, modified A base supported by at least 20 modified transcripts were identified as modified m6A sites. Nanom6A was developed to train, identify, and visualize the m6A of DRS reads, which is available at https://github.com/gaoyubang/nanom6A.*

2. There have been two papers reporting the same strategy of applying MazF to sequence a small set of m6A sites in m6ACA context. Estimated modification fractions were obtained. The authors need to at least validate their method based on the known results.

*Response: Thanks, we really appreciate this suggestion to validate our method. We totally agree with the suggestion because m6A-REF-seq (Zhang et al., 2019) or MAZTER-seq (Garcia-Campos et al., 2019) provide single base resolution for m6A sites in ACA motif. As suggested, we added newly-generated m6A-REF-seq (Fig. 4e) to validate our method based on direct RNA sequencing in Populus trichocarpa. We performed m6A-REF-seq in Populus trichocarpa following previous m6A-REF-seq libraries construction methods (Zhang et al., 2019). Then, m6A-REF-seq libraries were sequenced using the Illumina Nova6000 platform (Illumina, USA). We added following description in the revised manuscript: Recently, m6A-REF-seq (Zhang et al., 2019) or MAZTER-seq (Garcia-Campos et al., 2019) provide single-base resolution for m6A sites in ACA motif. To further validate the reliability of Nanom6A, we also performed m6A-REF-seq to validate the method based on direct RNA sequencing. We firstly perform m6A-REF-seq in Populus trichocarpa following previous m6A-REF-seq libraries construction methods (Zhang et al., 2019). Then, m6A-REF-seq libraries were sequenced using the Illumina Nova6000 platform (Illumina, USA) (Fig. 4e). We found the enrichment of ACA motif at the end of sequencing reads and more than 60% left and right reads from Illumina pair-end model began and terminated at ACA site, respectively (Fig. 4f). Our results on percentage of ACA at the end of sequencing reads are consistent with a previous study (Garcia-Campos et al., 2019), suggesting the high reliability of m6A-REF-seq. The m6A sites were identified using previously published scripts with default parameters (Garcia-Campos et al., 2019). As expected, the m6A sites in ACA motif based on our m6A-REF-seq were enriched near the stop codon (Fig. 4g). In total, m6A-REF-seq validated 80% m6A sites based on Nanom6A (Fig. 4h).*

*We have added the Figure 4e, f, g, h and the text accordingly in the revised manuscript.*

Tao Pan lab has reported modification fraction of a few sites using SCARLET. Those sites need to be validated.

*Response: Thank you for pointing this out to further validate our method using known m6A sites. As suggested, we added following new validation using reported modified sites using SCARLET:*

*To further evaluate the reliability of our method, we calculate all known m6A sites based on SCARLET (Liu et al., 2013). In total, the m6A modification of two human lncRNAs (MALAT1 and TUG1) and three human mRNAs (TPT1, ACTB, and BSG) have been reported (Liu et al., 2013), which presented reliable known modification sites from transcripts in vivo. We compared the Nanom6A identified m6A sites based on DRS data (Lorenz et al., 2020) with known m6A modification based on SCARLET method (Liu et al., 2013). Among two lncRNAs (MALAT1 and TUG1), MALAT1 (NR_002819.2) was supported by only three DRS reads, which were too few to cover known m6A sites. Thus, we excluded the comparison of MALAT1 from downstream analysis. In TUG1 RNA, Nanom6A identified ten known modified sites, which included site in 1114, 2601,2497, 2564, 2953, 3071, 5125, 5133, 5138 and 5910 and were consistent with the SCARLET method (Fig. 2d). Especially, we found that these sites located near the 3’ terminal region included more DRS reads support. For TPT1 mRNA (NM_003295.1), Nanom6A based DRS reads also presented consistent results with SCARLET in 687, 694 and 703 modified sites. ACTB mRNA (NM_001101.5) and BSG mRNA (NM_198591.1) have three known m6A modifications based on SCARLET method (Liu et al., 2013). Our method supported these modified sites and was consistent with SCARLET results (Fig. 2e, f). Notably, two modified sites showed high modification ratio based on Nanom6A using DRS data at 1216 in ACTB and 1335 in BSG, respectively. Thus, we selected these m6A modified sites for qualitative comparison between wild-type and METTL3 knockdown (shMETTL3). Using Nanom6A, the modification ratio at 1216 site from ACTB was 61.6% and 33.1% in wild-type and shMETTL3, respectively (Fig. 2e). The modification ratio at 1335 site from BSG transcripts was 60% and 47% in wild-type and shMETTL3, respectively (Fig. 2 f). Published MeRIP-Seq dataset (Meyer et al., 2012; Schwartz et al., 2014) also reported a similar decreasing trend, which further validated the result based on Nanom6A.*

*We have added Figure 2b, c, d, e, f and the text accordingly in the revised manuscript.*

3. More experimental and computational detailed are required in order to analyze the claims by the authors.

*Response: We appreciate this comment. As suggested, we now make this clear in the Methods section. More experimental details have been added in libraries constructions for Nanopore direct RNA sequencing and MeRIP-seq. Also, more Bioinformatics analysis details were added in method part and result part accordingly.*

Reviewer #2: The manuscript by Gao and Liu et al (2020) applied an XGBoost model to identify m6A modified bases (Nanom6A) in direct RNA nanopore sequencing (dRNS) data. The authors compared their XGBoost model against two previously published methods, EpiNano[1] and MINES[2], using previously published Arabidopsis dRNAS data[3]. The study claims that the number of m6A sites identified by Nanom6A is comparable to MINES. Further, the authors claim that Nanom6A pipeline is superior because it uses the raw ionic current information that is extracted using Tombo. I liked that the authors looked at the distribution of m6A across transcripts and on the poly(A) tail. These are worthy endeavors especially for studying plant biology.

*Response: Thank you for your appreciation of the submitted work. We have revised our manuscript according to your suggestions, as described below.*

Major comments:

The department of Biology and Program in Cell and Molecular Biology, Colorado State University, Fort Collins, CO, United States is not attributed to any author.

*Response: We apologize for the error. Colorado State University is attributed to our coauthor Anireddy S. N. Reddy. We corrected this in the revised manuscript.*

Lines 56-57: "other known RRACH (D=G/A, H=A/U/C) motif" Should the parentheses contain R=G/A?

*Response: As suggested we added R=G/A in the parentheses in the revised manuscript.*

The principle of using ionic current signals to detect modified RNA nucleotides has been described in several studies, including Smith et al[4], Workman et al[5], Ding et al[6], Lorenz et al[2]. Additionally, MINES[2] uses the same Tombo resquiggle function that is used by the authors in this study. These claims should be toned down, and appropriate citations should be included.

*Response: Thanks for the suggestion. As suggested, we revised this part and added appropriate citations as described below. It is intuitive to use ionic current signals from the sequencer to identify the base modification. Recently, several studies adopted this principle. For example, Gaussian mixture model has been reported to determine unsupervised modification number (Ding et al., 2020). Another study on 16S ribosomal RNA reports the correlation between base-call error and ionic current deviations from direct nanopore sequencing (Smith et al., 2019). In vitro transcribed RNAs in GGACU motif also further validated the correlation between m6A modification and ionic current (Workman et al., 2019). MINES also built motif-specific models based on pore current value (Lorenz et al., 2020). These pioneering studies proved that de novo identification of m6A is feasible. In this study, we also used ionic current signals directly and developed an XGBoost model for identification of m6A modification at a single-base resolution at an individual transcript level, which is necessary for estimation of m6A abundance. The revised manuscript included these references and description accordingly.*

Was a single Nanopore sequencing experiment sufficient to capture the transcriptome? Would the robustness of the data improve with replicates? And more sequencing should improve depth for low abundance transcripts?

*Response: According to your suggestion, we added biological repetition to improve the depth in the revised version using direct RNA sequencing kit (SQK-RNA002) on MinION platform, which presented 2-fold depth than the first biological repeat using direct RNA sequencing kit (SQK-RNA001). Through two biological repeats using different sequence platform (GridION and MinION) and different kits (SQK-RNA001 and SQK-RNA002), the Pearson's correlation coefficient of 12,087 overlapped m6A sites was 0.96 (Fig. 3c), which revealed that Nanom6A provides good reproducibility between biological repeats even when we used different libraries preparation kits and different sequencers. In total, 98% m6A sites from the first repeat overlapped with the second repeat, suggesting high reproducibility between two independent biological repeats (Fig. 3d). Due to higher sequence depth, the second repeat identified 17,293 novel m6A sites, which were missed in the first repeat. Obviously, these repeat2 specific modified sites mainly originated from low abundance transcripts (Fig. 3e), which suggested that improving the depth could increase the chance to identify m6A sites. We added Figure 3b, c, d and text accordingly in the revised manuscript.*

Albacore basecaller has been deprecated by ONT for over a year. Why did the authors choose it in place of Guppy which has superior performance? The data analyses and inferences would substantially improve with re-basecalling using Guppy RNA flip-flop model.

*Response: Thank you for the suggestion. As you suggested, we compared Albacore basecaller and Guppy. There were 552,021 and 725,156 reads generated from Albacore and Guppy, respectively. Thus, we could obtain more mapped reads for downstream analysis. After mapping these reads using minimap2 we found the error rate for Albacore and Guppy was 1.893623e-01 and 1.168010e-01, respectively. Thus, Guppy presented superior performance and obviously improved the accuracy during base calling. In the revised version, we updated all the results accordingly based on Guppy according to the suggestion. We updated all the text and figures accordingly in the revised manuscript based on this new method.*

The authors did not compare Nanom6A against the 17,491 m6A sites from the Parker et al study[3]. This is an important comparison to be performed since a lot of Nanom6A development used those data.

*Response: Thank you for this suggestion. We used 17,491 sites, which were named as differential error sites (DES) in Parker et al study (Parker et al., 2020b). We excluded non-RRACH motifs and obtained 4,936 m6A sites with RRACH motif for downstream comparison. We further compared 17,491 differential error sites (DES) from Parker et al study (Parker et al., 2020a), which included a total of 4,936 sites with RRACH motif. About 66% (3,289/6,936) RRACH motifs from Parker et al study (Parker et al., 2020a) also reported by Nanom6A (Fig. 2i). We provided a detailed description of this analysis in the text accordingly in the revised manuscript.*

Also, the comparisons with other methods (EpiNano and MINES) needs to be performed one at a time (Nanom6A vs. EpiNano for all motifs, and Nanom6A vs. MINES for the four motifs). The claim that Nanom6A can identify m6A in context independent motifs needs to be substantiated.

*Response: As suggested, we performed the comparisons of Nanom6A vs. EpiNano (Fig. 3 j, k, l) and Nanom6A vs. MINES (Figure 3 m, n, o) one at a time. Venn diagram between Nanom6A vs. EpiNano depicted the overlap of 2,326 m6A sites, 10,012 Nanom6A specific and 2,838 EpiNano specific sites (Figure 3j), respectively. The distribution of m6A sites showed that 2,326 overlapped and 10,012 Nanom6A specific m6A sites were more enriched near stop codon and 3’UTR region than that of 2,838 EpiNano specific sites (Fig. 3k). Especially, we found that the ratio of m6A from 10,012 Nanom6A specific sites was lower than that of 2,326 overlapped sites (Fig. 3l), which suggested that Nanom6A has an advantage in detecting low m6A/A ratio since Nanom6A could detect m6A at single transcript resolution. MINES only generated m6A sites for AGACT, GGACA, GGACC, and GGACT. Thus, we only compared the above four motifs using these three methods with MINES. The comparison between Nanom6A vs. MINES (Fig. 3m, n, o) also presented a similar trend with Nanom6A vs. EpiNano. We updated the text accordingly in the revised manuscript.*

The authors claim that Nanom6A identified 27% of m6A sites uniquely in comparison with EpiNano and MINES. Using the numbers from Figure 2c, a total of 55,213 m6A sites were identified. There were only 2,317 that were only identified by Nanom6A. That's only 4.1%. If we eliminate the EpiNano counts, there are 13,389 m6A sites identified by MINES and Nanom6A. Those 2,317 Nanom6A sites now account for ~17% of the total, still not 27%. How are they calculating the percentage of Nanom6A only sites? This needs clarity and a better explanation.

*Response: In the first version, the percentage of 27% was calculated based on the following formula: 2317/(2317+2893+2555+796). We used the total number from Nanom6A as denominator. In this revised manuscript, according to your suggestion, we divided three-way comparison into pair-wised comparison: Nanom6A vs. EpiNano for all motifs (Fig.3j), and Nanom6A vs. MINES for the four motifs (Fig. 3m). We updated the text accordingly in the revised manuscript.*

Why is this three-way comparison of techniques not established before applying Nanom6A to Populus trichocarpa SDX. It is important to demonstrate and compare Nanom6A on Arabidopsis data, followed by applying it on the dRNS data collected for P. trichocarpa.

*Response: As suggested, we added the three-way comparison in Arabidopsis. For AGACT, GGACA, GGACC, and GGACT motifs, about 40% (1,215/3,055) m6A sites detected with Nanom6A method overlapped with MINES or EpiNano (Fig. 2h). We updated the text accordingly in the revised manuscript.*

The authors performed one MeRIP-Seq experiment. Are replicates needed/useful?

Would higher sequencing depth for MeRIP-Seq resolve issues with low abundance transcripts?

*Response: According to your suggestion, we added a biological replicate of MeRIP-Seq in the revised manuscript. The overlapped m6A sites from two repeats presented higher coverage than that of novel m6A sites from the second repeat, which suggested higher sequencing depth for MeRIP-Seq could detect more m6A sites from low abundance transcripts (Fig. 4c). We updated the text accordingly in the revised manuscript.*

Figure 2 is busy and needs simplification. For example, panels 2d and 2e could be moved to the supplementary section. Each panel needs proper explanation. E.g. 2e computes log for number of reads. This really needs to be the raw counts (or raw counts + normalized proportions). 2b and 2c can be a table. Line 138: What r value is being calculated for MeRIP-seq and Nanom6A? An r value of 0.53 seems low for trying to precisely predict m6A sites in P. trichocarpa.

*Response: As suggested, we now changed the previous two figures into five figures in the revised manuscript to avoid overcrowded figures. We recognize that MeRIP-Seq and DRS belong to two totally different methods, which caused the low r value. In the revised version, we used the data based on direct RNA sequencing from wild-type and defective m6A writer complex component in both plant and mammal to validate the qualitative profiling of Nanom6A to address your question. Firstly, we used vir-1 and VIRc from Parker study (Parker et al., 2020b) to validate the quantitative analysis using Nanom6A. The m6A ratios from vir-1 were lower than that from VIRc, which showed the ability of Nanom6A in qualitative profiling (Fig. 2k). We further identified RNA modification using Nanom6A based on direct RNA sequencing data (FAST5 files) from wild-type and METTL3 knockdown (shMETTL3) (Lorenz et al., 2020) to identify m6A sites using Nanom6A, which showed that both modification ratio (Fig. 2b) and number of m6A sites (Fig. 2c) were decreased in METTL3 knockdown sample. Thus, depletion in both vir-1 and shMETTL3 partly supported the qualitative profiling of Nanom6A. Thus, we removed Figure 2g (of the original version) and added above two additional analyses (Fig. 2k and Fig. 2b) to validate the qualitative profiling of Nanom6A. We updated the text accordingly in the revised manuscript.*

Line 157: Is there a poly(A) tail length difference between reads using a canonical poly(A) site and an alternative poly(A) site?

*Response: Thank you very much for raising this interesting question since Nanopore direct RNA sequencing has a great advantage in detecting poly(A) length, which has been reported in Arabidopsis (Parker et al., 2020b), in vitro transcribed RNAs (Krause et al., 2019), and GM12878 cells (Workman et al., 2019). In this study, we adopted a standard library preparation protocol for native RNA sequencing, which retained the full poly(A) tail to identify isoform-specific poly(A) tail length. We identified poly(A) tail length with a nanopolish-polya module (Ding et al., 2020) in the nanopolish package (0.11.1). We found that the mean and median poly(A) length for poplar mRNA was 92nt and 82nt, respectively (Fig. 5a). The gene expression and poly(A) tail length showed a negative correlation (r = -0.26) in Populus (Fig. 5b), which was consistent with the previous study (Parker et al., 2020b). In total, there were 2421 alternative polyadenylation genes included proximal and distal poly(A) sites, which showed two-fold change in poly(A) length (Fig. 5c). For example, PARVUS-L-2 is galacturonosyltransferase-like 1, which shows tissue-specific expression in stem-derived developing xylem (Kim et al., 2019). Our DRS data showed that this secondary wall biosynthetic gene included two polyadenylation sites with different poly(A) length (Fig. 5d). The transcripts with distal polyadenylation presented longer poly(A) than that of proximal transcripts. This analysis provided preliminary poly(A) tail lengths from alternative polyadenylation to investigate the potential regulatory role of poly(A) tails in degradation and translation. We added the text accordingly in the revised manuscript.*

Line 178: I'm not convinced that the authors have achieved m6A identification of individual transcripts without evaluating a sample with known m6A to non-m6A ratios. At minimum, the authors need to perform and include control experiments, and tone down the claims about quantification.

*Response: Thanks for your constructive suggestion. As suggested, we used synthesized mRNAs from previous study (Liu et al., 2019), which included two known types of reads: modified and unmodified reads. Firstly, we used the Nanom6A model to predict known modified and unmodified reads, respectively. In total, 91%-96% of the modified and unmodified sites from individual transcripts could be successfully identified (Fig. 1c, d). Secondly, we mixed known modified and unmodified reads together to simulate datasets with different m6A to non-m6A ratios, which covered the whole interval of modified ratio from 0 (all unmodified DRS reads) to 1 (all modified reads). The above simulated DRS dataset was analyzed by Nanom6A, which showed that the predicted m6A to non-m6A ratios based on Nanom6A presented a strong correlation with known m6A to non-m6A ratios (Fig. 1e). We also simulated different sequence depth to generate three different levels by mixing 20, 50 and 100 DRS reads in 10000 times for each different m6A to non-m6A ratio, respectively. These results show that higher depth could generate a higher correlation (Fig. 1e). We added the text accordingly in the revised manuscript.*

Line 204: Which reverse transcriptase was used for first strand synthesis? Did the authors follow standard protocol recommended by ONT?

*Response: SuperScript III reverse transcriptase (ThermoFisher SCIENTIFIC-18080093) was used for first strand synthesis (Fig. 3a). It should be noted that the motor protein was linked to the RNA strand, so that the RNA was sequenced to detect RNA modification, and not the first-strand cDNA, which was merely present to reduce or eliminate RNA secondary structures to ensure efficient RNA strand translocation through the Nanopore (Soneson et al., 2019). We updated the method “Construction of Library and RNA Direct Sequencing” accordingly in the revised manuscript. We also added Figure 3a to address this question.*

The motivation for using the XGBoost model was never described by the authors. I appreciate it is an ensemble method, but it is important to explain why it could be applied to Nanopore ionic current data.

*Response: Thanks for point this out. Before we used XGBoost model for Nanom6A, we compared XGboost with several other classification algorithm, the results showed that XGBoost presented the best performance. Thus, we used XGBoost model in this study. We updated Figure 1b (of revised version) and the text accordingly in the revised manuscript.*

The Nanom6A pipeline code is not open source. The authors provide a Docker container but not the code itself. I can understand if there is a reason for this, but it was not provided in the manuscript. Also, the SRR8491764 accession is not public so I could not download the data.

*Response: Thanks. We really appreciate your constructive suggestions. We revised our manuscript according to the suggestions. Now we deposited the source code of Nanom6A in GitHub (https://github.com/gaoyubang/nanom6A).*

*According to your suggestion, we released the raw data of DRS. The raw signal file can be downloaded from following hyperlink directly: https://sra-download.ncbi.nlm.nih.gov/traces/sra68/SRZ/012822/SRR12822922/Ptr-WT-SDX-20200827.tar*

Minor comments:

The manuscript needs editing. There are problems with grammar and word use.

Some examples are below

Line 25 - omit "conservative" replace with "conserved"

Line 27 - Replace in single-base with "at the single base level"

Line 30 - add the word "an" before XGBoost model

Line 38 - replace "in" with "for"

Line 65 - omit distinct, insert "distinguish"

Line 254 - The remaining 1/10... (sentence doesn't make sense)

Figure 1 - spelling separate not seperate

Overall, the authors present an interesting manuscript that could be improved.

*Response: We apologize for the grammatical and typographical errors in the submitted manuscript. We corrected these in the revised version.*

*Reference*

*Ding, H., Bailey, A.D., Jain, M., Olsen, H., and Paten, B. (2020). Gaussian Mixture Model-Based Unsupervised Nucleotide Modification Number Detection Using Nanopore Sequencing Readouts. Bioinformatics.*

*Garcia-Campos, M.A., Edelheit, S., Toth, U., Safra, M., Shachar, R., Viukov, S., Winkler, R., Nir, R., Lasman, L., Brandis, A., et al. (2019). Deciphering the “m6A Code” via Antibody-Independent Quantitative Profiling. Cell 178, 731-747.e716.*

*Kim, M.-H., Cho, J.-S., Jeon, H.-W., Sangsawang, K., Shim, D., Choi, Y.-I., Park, E.-J., Lee, H., and Ko, J.-H. (2019). Wood transcriptome profiling identifies critical pathway genes of secondary wall biosynthesis and novel regulators for vascular cambium development in populus. Genes 10, 690.*

*Krause, M., Niazi, A.M., Labun, K., Cleuren, Y.N.T., Müller, F.S., and Valen, E. (2019). tailfindr: alignment-free poly (A) length measurement for Oxford Nanopore RNA and DNA sequencing. RNA 25, 1229-1241.*

*Lindenbaum, P. (2015). JVarkit: java-based utilities for Bioinformatics. FigShare, doi 10, m9.*

*Liu, H., Begik, O., Lucas, M.C., Ramirez, J.M., Mason, C.E., Wiener, D., Schwartz, S., Mattick, J.S., Smith, M.A., and Novoa, E.M. (2019). Accurate detection of m 6 A RNA modifications in native RNA sequences. Nature communications 10, 1-9.*

*Liu, N., Parisien, M., Dai, Q., Zheng, G., He, C., and Pan, T. (2013). Probing N6-methyladenosine RNA modification status at single nucleotide resolution in mRNA and long noncoding RNA. Rna 19, 1848-1856.*

*Lorenz, D.A., Sathe, S., Einstein, J.M., and Yeo, G.W. (2020). Direct RNA sequencing enables m6A detection in endogenous transcript isoforms at base-specific resolution. RNA 26, 19-28.*

*Meyer, K.D., Saletore, Y., Zumbo, P., Elemento, O., Mason, C.E., and Jaffrey, S.R. (2012). Comprehensive analysis of mRNA methylation reveals enrichment in 3′ UTRs and near stop codons. Cell 149, 1635-1646.*

*Parker, M.T., Knop, K., Sherwood, A.V., Schurch, N.J., Mackinnon, K., Gould, P.D., Hall, A.J., Barton, G.J., and Simpson, G.G. (2020a). Nanopore direct RNA sequencing maps the complexity of Arabidopsis mRNA processing and m6A modification. eLife 9.*

*Parker, M.T., Knop, K., Sherwood, A.V., Schurch, N.J., Mackinnon, K., Gould, P.D., Hall, A.J., Barton, G.J., and Simpson, G.G. (2020b). Nanopore direct RNA sequencing maps the complexity of Arabidopsis mRNA processing and m6A modification. Elife 9, e49658.*

*Schwartz, S., Mumbach, M.R., Jovanovic, M., Wang, T., Maciag, K., Bushkin, G.G., Mertins, P., Ter-Ovanesyan, D., Habib, N., and Cacchiarelli, D. (2014). Perturbation of m6A writers reveals two distinct classes of mRNA methylation at internal and 5′ sites. Cell reports 8, 284-296.*

*Smith, A.M., Jain, M., Mulroney, L., Garalde, D.R., and Akeson, M. (2019). Reading canonical and modified nucleobases in 16S ribosomal RNA using nanopore native RNA sequencing. PloS one 14, e0216709.*

*Soneson, C., Yao, Y., Bratus-Neuenschwander, A., Patrignani, A., Robinson, M.D., and Hussain, S. (2019). A comprehensive examination of Nanopore native RNA sequencing for characterization of complex transcriptomes. Nat Commun 10, 3359.*

*Workman, R.E., Tang, A.D., Tang, P.S., Jain, M., Tyson, J.R., Razaghi, R., Zuzarte, P.C., Gilpatrick, T., Payne, A., and Quick, J. (2019). Nanopore native RNA sequencing of a human poly (A) transcriptome. Nature Methods 16, 1297-1305.*

*Zhang, Z., Chen, L.-Q., Zhao, Y.-L., Yang, C.-G., Roundtree, I.A., Zhang, Z., Ren, J., Xie, W., He, C., and Luo, G.-Z. (2019). Single-base mapping of m6A by an antibody-independent method. Science advances 5, eaax0250*.

**Second round of review**

**Reviewer 2**

Dear authors,

Thank you for addressing my comments. I do not have further questions. The manuscript has improved from its previous version. I will suggest that the authors try and attempt simplifying the figures to improve the readability of the manuscript.

Best wishes.
